# Determining the extent of maternal-foetal chimerism in cord blood

**DOI:** 10.1038/s41598-019-41733-w

**Published:** 2019-03-27

**Authors:** Rianne Opstelten, Manon C. Slot, Neubury M. Lardy, Arjan C. Lankester, Arend Mulder, Frans H. J. Claas, Jon J. van Rood, Derk Amsen

**Affiliations:** 1 Sanquin Research, Dept of Hematopoiesis, Amsterdam, The Netherlands, and Landsteiner Laboratory, Amsterdam UMC, University of Amsterdam, Amsterdam, The Netherlands; 2 Sanquin Diagnostics BV, Department of Immunogenetics, Amsterdam, The Netherlands; 3 Willem-Alexander Children’s Hospital, Leiden University Medical Center, Leiden, The Netherlands; 4 Department of Immunohematology and Blood Transfusion, Leiden University Medical Center, Leiden, The Netherlands

## Abstract

During pregnancy, maternal T cells can enter the foetus, leading to maternal-foetal chimerism. This phenomenon may affect how leukaemia patients respond to transplantation therapy using stem cells from cord blood (CB). It has been proposed that maternal T cells, primed to inherited paternal HLAs, are present in CB transplants and help to suppress leukaemic relapse. Several studies have reported evidence for the presence of maternal T cells in most CBs at sufficiently high numbers to lend credence to this idea. We here aimed to functionally characterise maternal T cells from CB. To our surprise, we could not isolate viable maternal cells from CB even after using state-of-the-art enrichment techniques that allow detection of viable cells in heterologous populations at frequencies that were several orders of magnitude lower than reported frequencies of maternal T cells in CB. In support of these results, we could only detect maternal DNA in a minority of samples and at insufficient amounts for reliable quantification through a sensitive PCR-based assay to measure In/Del polymorphisms. We conclude that maternal microchimerism is far less prominent than reported, at least in our cohort of CBs, and discuss possible explanations and implications.

## Introduction

Eutherian pregnancy creates a challenge for both the maternal and foetal immune system. Foetuses express minor and major histocompatibility antigens inherited from their fathers (Inherited Paternal Antigens; IPA), which are foreign to the maternal immune system. Vice versa, the maternal antigens that are *not* inherited by the foetus (Non-Inherited Maternal Antigens; NIMA) are foreign to the foetal immune system. Curiously, it has been shown that there is passage of maternal cells into the child across the placenta and vice versa during pregnancy. This phenomenon makes it impossible for the two immune systems to simply ignore one another. Given the intimate connection between the maternal and foetal circulation in the placenta, mechanisms must be in place to avoid immune-mediated rejection by and of the foetus. Indeed, the placental environment seems highly conducive to induction of tolerance^1^. Tolerance is not absolute, however. This is shown, for instance, by the appearance of antibodies and cytotoxic T cells against IPA in multiparous women^2,3^ as well as the presence of antigen-experienced foetal T cells directed against NIMA in cord blood (CB)^4^. Mother and child thus apparently leave an immunological imprint on one another. Interestingly, epidemiological studies have indicated that this imprint may affect the outcome of stem cell transplantation using CB grafts, which are used in patients with a broad range of both malignant and non-malignant diseases^5^. It was argued by van Rood *et al*., that maternal T cells inside the foetus (Maternal Microchimerism; MMc) are sensitized to the IPA of the child during pregnancy. Upon transfer into patients, such sensitized maternal T cells might then generate efficient anti-leukaemic responses in the CB recipient, if this recipient expresses the same IPA HLA^6^. Consistent with this hypothesis, leukaemia patients that had one or more HLAs in common with their CB donor’s IPA had a significantly lower tumour relapse rate than those that had no such match.

If matching for IPA HLAs between donor and patient indeed improves treatment success, it would make sense to specifically select CBs for this criterion before transplantation into leukaemia patients. Confidence in the validity of the concept behind IPA-matching would be much enhanced if the presence of sensitized maternal T cells in CB could be confirmed. According to a prominently published study, remarkably high frequencies of maternal T cells can be found in most CBs by FACS (up to 1% of the total T cell pool)^7^, which supports the idea that such cells could have a significant role after transplantation into patients. Similarly high frequencies were also found in some other studies, although the degree of chimerism reported varied over several orders of magnitude between and even within studies^8–15^.

Given the important implications for clinical practice, we here set out to examine whether maternal T cells in CB might exhibit signs of sensitisation to IPA HLA. To our surprise, given the high frequencies reported by Mold, *et al*.^7^, we could not detect intact, viable maternal T cells using FACS-based methods. Such cells were not even found after using state-of-the-art enrichment techniques that were sufficient to isolate T cells mixed into heterologous samples at frequencies lower than those reported for maternal T cells in CB. Using a sensitive PCR-based method, we found that maternal DNA was only detectable in a minority of CB samples (17.9%), in agreement with other studies^8,10,11^. Furthermore, even when maternal DNA could be detected, the proportion was much lower than suggested previously^7^ and no intact maternal cells could be found. Finally, we highlight some pitfalls that may lead to false positives when using anti-HLA antibodies to assess MMc by FACS, which may underlie some of the differences reported regarding the prevalence of MMc.

Based on our results, we conclude that maternal T cells are present in CB (at least in our cohort of CBs tested) in much lower numbers than reported^7^.

## Results

### Sample storage leads to asymmetric loss of HLA expression

**Figure 1:**
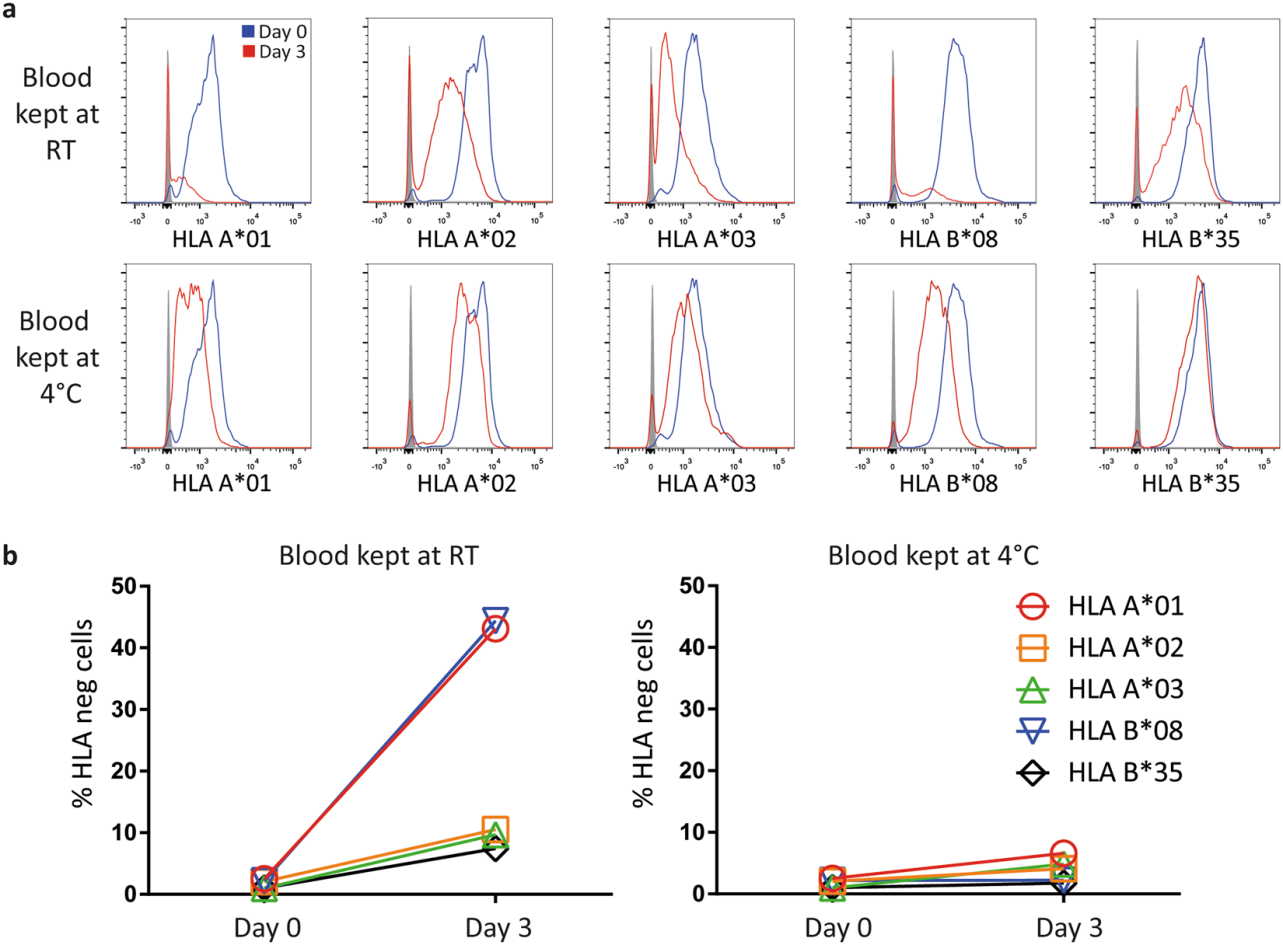
Storage at RT leads to asymmetric loss of HLA expression. Whole blood was either kept at room temperature (RT) or at 4 °C for 3 days. Blood was Ficolled on the day of the HLA staining and FACS analysis. (**a**) Histograms per anti-HLA antibody are shown comparing the staining on Day 0 (blue) to that on Day 3 (red). (**b**) Percentage of cells that are negative for the HLA staining per day (see key in figure for symbols used).

The easiest way to distinguish between maternal and foetal cells by flow cytometry is through the use of antibodies to HLA molecules that discriminate between mother and child. For practical reasons, there is often a delay between harvesting of the CBs and their delivery to the lab. During this time, CBs are often kept at room temperature (RT). To determine whether these conditions may affect cell surface expression of HLA molecules, we stored peripheral blood from volunteer donors in the collection tubes at RT or at 4 °C and isolated peripheral blood mononuclear cells (PBMCs) immediately before HLA serotyping at different time points. At RT, up to 44,4% of the lymphocytes lost surface expression of some HLAs completely, and on the remaining cells, the intensity of the staining was greatly diminished (Fig. 1a). This effect was also apparent when cells were stored at 4 °C, albeit to a lesser extent. For instance, expression of HLA-A*01 was almost completely lost on around half the cells stored at RT after 3 days and profoundly diminished in intensity even at 4 °C, whereas expression of HLA-B*35 was more stable, especially at 4 °C (Fig. 1). Loss of HLA staining was found using antibodies to multiple HLA molecules and when different antibodies to the same HLA molecules were used (see Supplementary Fig. S1), suggesting that this phenomenon is not caused by changes in specific epitopes, but rather by loss of surface expression. This is a problem for reliable detection of (small numbers of) maternal T cells in CB. It can make maternal T cells indistinguishable from foetal cells, if discrimination is based exclusively on an HLA allele expressed by the mother but not by the child. Perhaps more problematic, foetal cells may be mistaken for maternal cells if discrimination is based on a single HLA difference expressed by the child but not by its mother. These findings show that FACS-based identification of maternal cells in CB can only be performed reliably by using a positive identifier HLA expressed on maternal but not infant cells and has the caveat that the percentage of maternal cells may be underestimated. Finally, long delays between harvesting and analysis should be avoided.

### Failure to detect a clear population of maternal cells in CB by FACS

**Figure 2:**
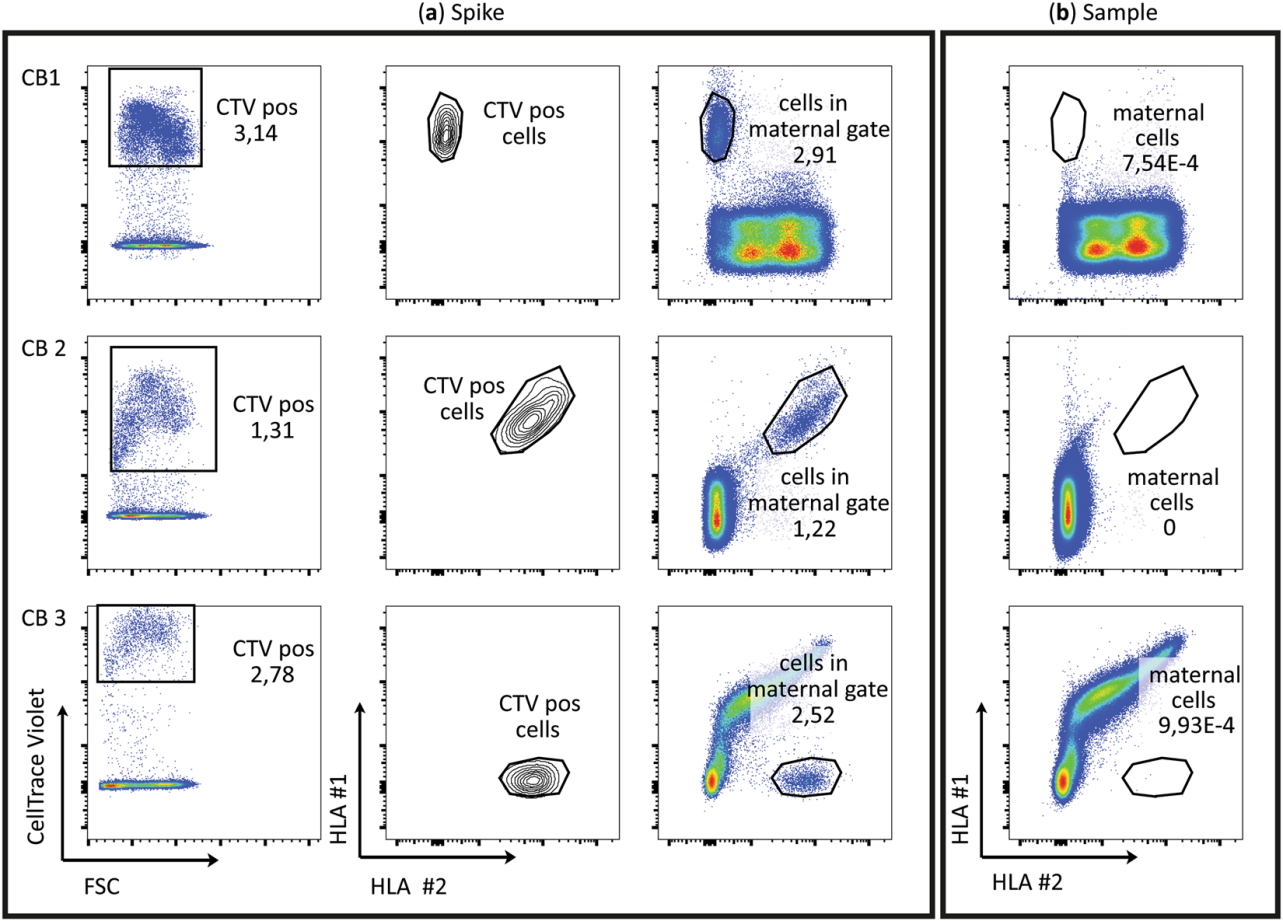
Identification of maternal cells in three different CB samples on FACS. (**a**) A blood sample from the mother was labelled with CTV and spiked into a CB aliquot to establish the position of the maternal gate in the maternal and foetal HLA plots. (**b**) The lymphocyte gates of the unspiked CB samples are shown with the same HLA staining and the percentage of cells that fall into the maternal gate is reported.

**Figure 3:**
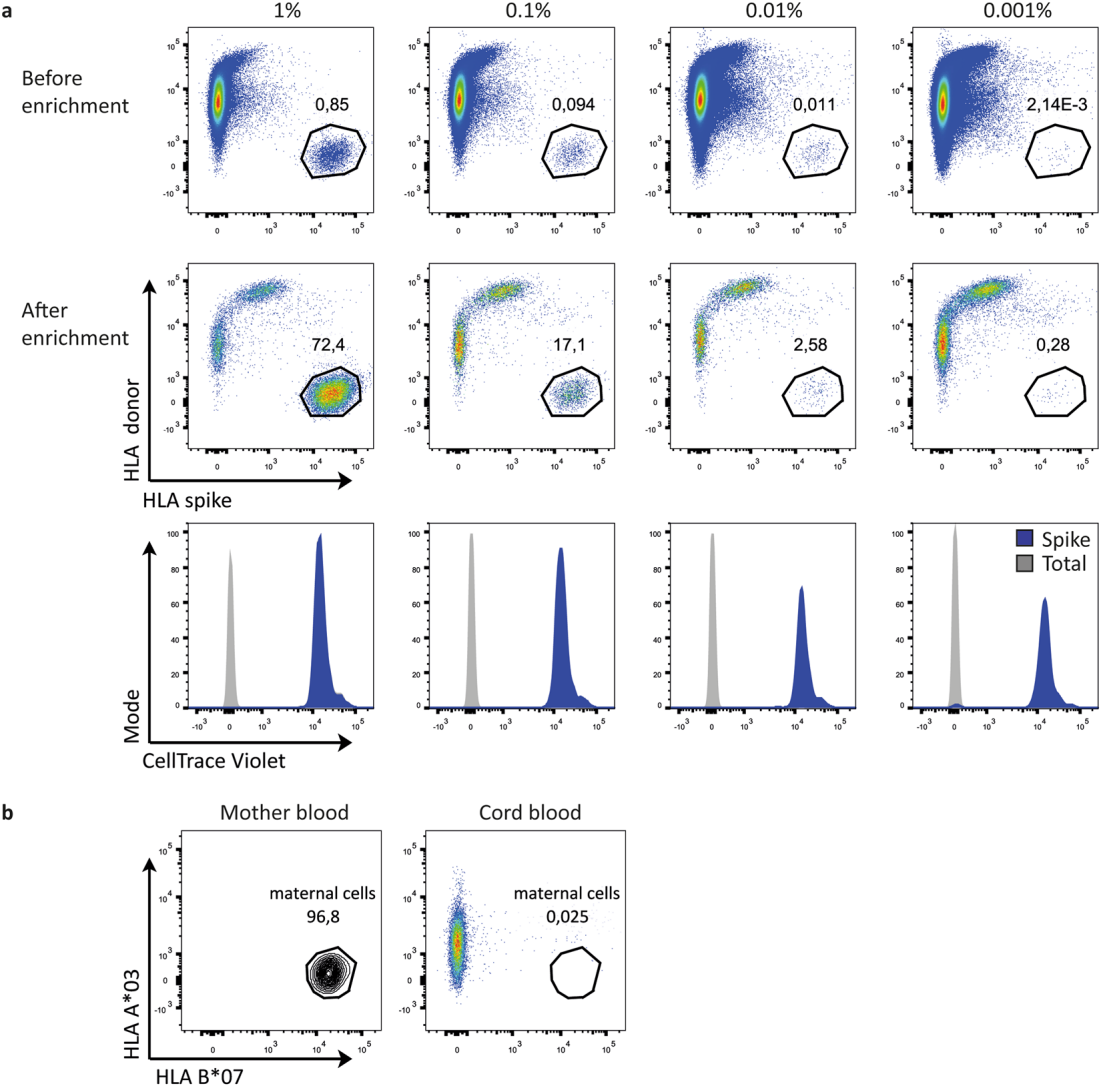
MACS enrichment of CB sample for maternal cells. HLA-based MACS enrichment was validated by spiking small quantities (1%, 0.1%, 0.01% and 0.001%) of CTV-stained PBMCs from one donor into another donor and FACstaining for the HLAs of both donors before (top) and after enrichment (middle). To validate that the enriched cells are indeed the spiked in cells, the CTV staining of the total lymphocytes (grey) and spiked gate (blue) are shown (bottom) (**a**). The same approach was used to enrich maternal cells (A*02/*02, B*07/*51) from a CB sample (A*02/*03, B*44/*51). FACS plots of the lymphocyte gate of the mother blood (left) and the enriched cord blood (right) are shown (**b**).

Taking these caveats into consideration, we searched for intact maternal cells in CB using flow cytometry. Child and mother were first serotyped by staining the samples with an extensive panel of anti-HLA antibodies. Next, in order to determine the proper location of the maternal cell gate, we stained a blood sample from the mother of each CB donor with CellTrace^TM^ Violet (CTV) and spiked it into an aliquot of CB. When gating for the CTV positive cells, we could see exactly where the maternal cells would be situated in the lymphocyte gate when plotting two HLAs against each other (Fig. 2). We then stained the (unspiked) bulk CB sample for the same HLAs and determined the percentage of cells in the maternal gate. We pursued different strategies to identify maternal cells, such as staining for the IPA HLA of the foetal cells and the NIMA HLA of the maternal cells (**CB1**). We also stained for two NIMA HLAs so that maternal cells would be in the double positive quadrant (**CB2**) or stained for one IPA and one IMA (inherited maternal antigen) HLA, so that the foetal cells were in the double positive quadrant, and the maternal cells in the single positive quadrant (**CB3**). We failed to detect any cells in the maternal gate in one of the three CBs examined (**CB2**) (Fig. 2b). Cells were detected in the maternal gate in the other two CBs, but at very low frequencies (<0.001%, the cut-off of the sensitivity of our FACS detection method Fig. 3a) that were 2 to 3 orders of magnitude lower than reported by Mold *et al*.^7^.

### T cells detected in the maternal gate are cells from the child

**Figure 4:**
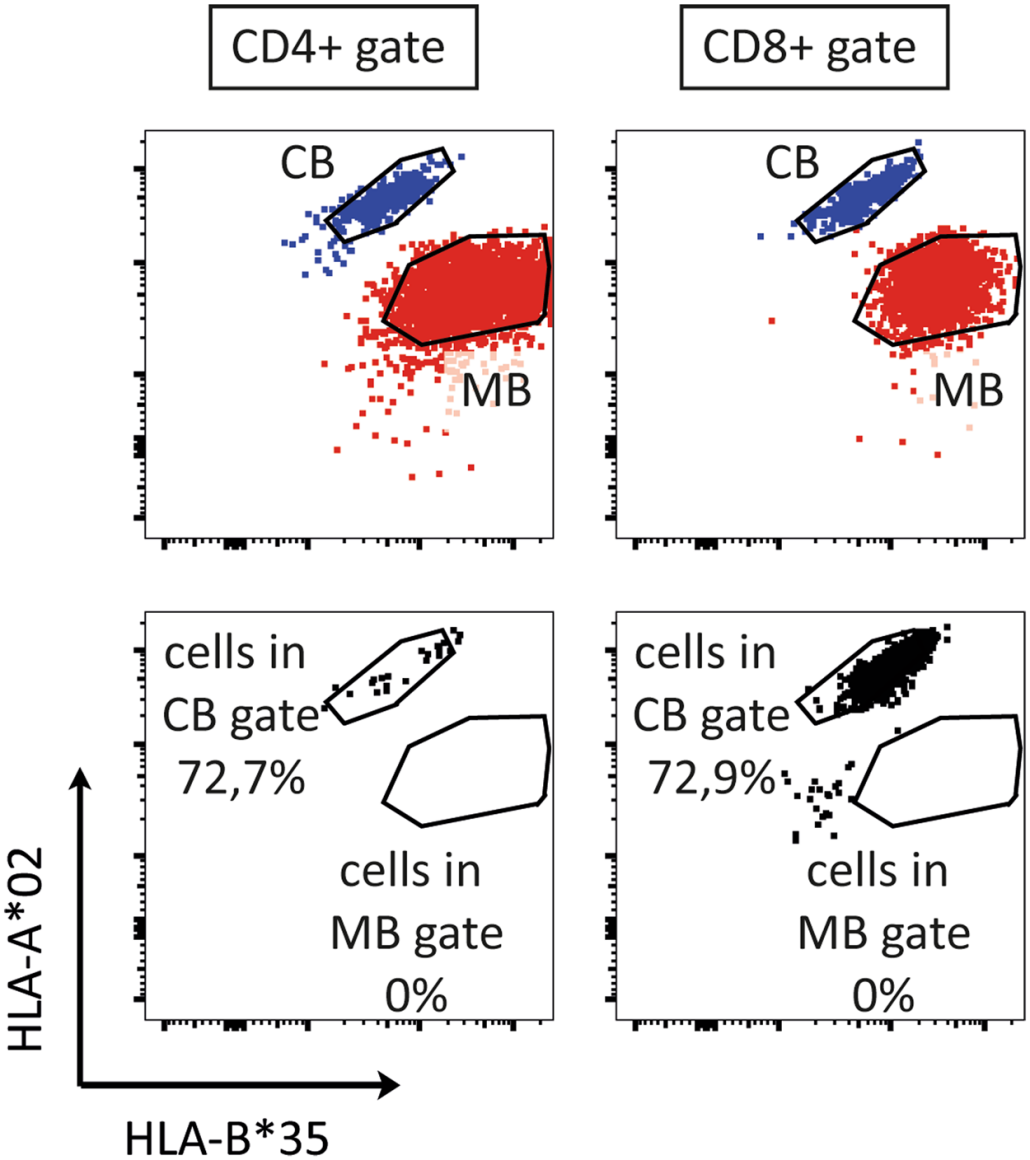
Culture of sorted cells from maternal gate of CB sample. All cells within the maternal gate were isolated by FACsorting and expanded for 24 days with anti-CD3, anti-CD28 and IL-2. The full CB and paired MB were also cultured to be able to determine the CB and MB gate after expansion. The position of the maternal gate was determined with a CTV stained spike of expanded MB into an expanded CB aliquot (top). The bottom graphs show the sorted cells from the maternal gate stained for the same HLAs gated both on CD4^+^ cells (left) and CD8^+^ cells (right) after expansion *in vitro*.

The numbers of maternal cells detected were much lower than the previously reported 0,1%^7^. It is difficult to know from these results, whether the few cells falling into the maternal gate in two of the three CB samples are truly of maternal origin, or whether these are a consequence of signal noise or of an asymmetric loss of HLA expression on a small number of foetal cells (in CB3). It has proven possible to enhance the reliability of detection of very low frequencies of cells by pre-enrichment using magnetic-activated cell sorting (MACS)^16^. We therefore established a MACS-based pre-enrichment protocol, again using spiked in cells. This protocol reliably afforded greater than two orders of magnitude enrichment of cells that were spiked in at frequencies down to 0.001% (Fig. 3a). However, when applying this protocol to CBs, a convincing population was not found in the maternal gate that was set based on the mothers’ blood (Fig. 3b) (events in gate: n = 3 cells; CBMC input before MACS: n = 10^8^ cells). This would argue that the few cells found in the maternal gate by FACstaining (Fig. 2b) may not be genuine maternal cells. As maternal cells could theoretically be lost preferentially during MACS procedures, we also FACsorted the cells in the maternal gate. We furthermore reasoned that the high complexity of the CB sample and the possible presence of dying cells might compromise the reliability of the signals. We therefore reduced this complexity and ensured optimal viability by expanding the selected cells *in vitro* after FACsorting. We cultured full CB and paired mother blood (MB) in parallel to be able to define the correct maternal cell gate after culture. We chose CB3 from Fig. 2, as this sample had the highest percentage of cells in the maternal gate. In total, we sorted out 1158 cells and cultured these cells for 24 days with anti-CD3, anti-CD28 and IL-2 to expand any T cells present. After 24 days, the cells were FAC stained for the IPA (HLA-A*02) and the IMA (HLA-B*35) and T cell markers. Figure 4 shows that CB (blue) and MB (red) CD4^+^ and CD8^+^ T cells can still clearly be distinguished based on IPA and IMA after 24 days of culture (top row). None of the FACsort-enriched cells from the original maternal gate (bottom row), however, fell into the MB gate after enrichment and expansion, showing that these are cells of the child and not of maternal origin.

### Screening for maternal DNA in CB shows that maternal DNA can only be found in a fraction of the CB samples and at low amounts

**Figure 5:**
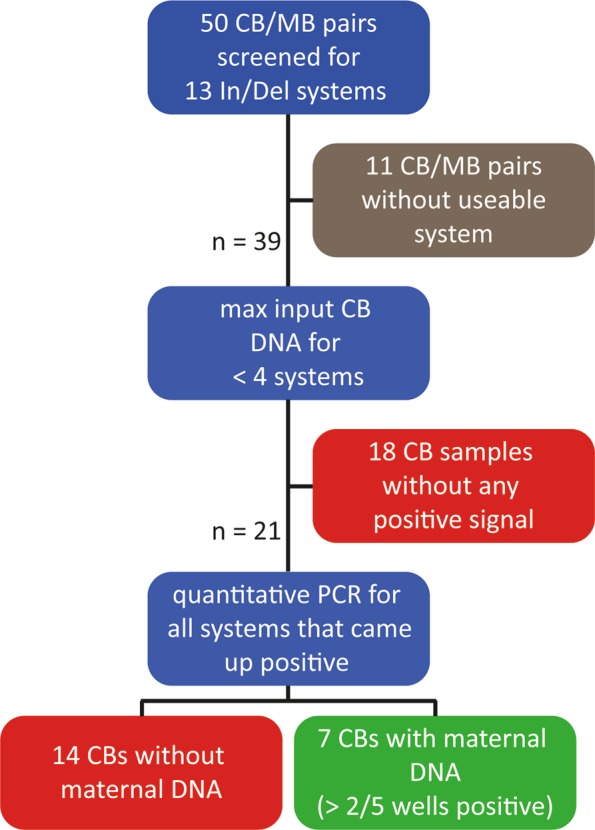
Workflow for the In/Del screening and detection of maternal DNA in 50 CB/MB pairs.

Since we could not unequivocally detect intact maternal cells in the CBs analysed by flow cytometry, we decided to screen for the presence of maternal DNA in our samples. For this, we used a real-time qPCR assay that screens for 13 In/Del polymorphisms that were designed to detect hematopoietic microchimerism after bone marrow transplantation^17^. We tested 50 CB/MB pairs in total (see Fig. 5 for the workflow). For the 39 CB/MB pairs with a system for which the mother was positive and the child was negative, we ran an initial screening with the maximum input of DNA (~500 ng) per CB sample to see if any of the systems came up positive in any of the wells tested (max 3 systems per CB). Twenty one out of the 39 CB samples had at least 1 well that came up positive for at least 1 system tested (Table 1).

**Table 1:** Results of screening with useable In/Del systems for each CB sample.

ID	Case no.	No. In/Del systems with pos. mother and neg. child	No. systems tested with 500 ng input DNA	No. systems that came up positive	No. wells that came up positive
389	1	1	1	0	
407	2	3	3	0	
411	3	2	2	1	1 of 3
417	4	2	2	0	
439	5	3	2	2	3 of 3
467	6	2	2	1	1 of 3
473	7	1	1	1	1 of 3
477	8	5	3	0	
483	9	2	2	0	
487	10	2	2	0	
491	11	3	2	0	
495	12	1	1	0	
506	13	3	2	1	2 of 3
510	14	2	2	0	
361	15	2	1	0	
513	16	1	1	1	3 of 3
517	17	4	3	1	2 of 3
535	18	1	1	0	
543	19	2	2	1	1 of 3
553	20	1	1	0	
579	21	1	1	1	3 of 3
836	22	3	3	2	1 of 3
846	23	2	2	0	
990	24	2	2	0	
133	25	3	3	1	1 of 3
137	26	2	2	2	1 of 3; 3 of 3
145	27	2	2	0	
155	28	1	1	1	1 of 3
177	29	1	1	0	
191	30	1	1	1	2 of 3
209	31	2	1	1	1 of 3
213	32	2	2	1	2 of 3
224	33	2	2	0	
238	34	2	2	1	1 of 3
245	35	2	2	1	2 of 3
253	36	3	3	1	1 of 3
273	37	2	2	1	1 of 3
301	38	1	1	0	
357	39	3	3	1	2 of 3

We then attempted to quantify the proportion of maternal DNA in each of these 21 CB samples. To this end, we ran 5 replicates of each sample with a maximum input of CB DNA for each system that came up positive in the screening, alongside a standard curve for each system tested. Seven CB samples tested positive for maternal DNA. Considering the sensitivity of each system, the percentage of maternal DNA was less than 0.1% (n = 2) or less than 0.01% (n = 5) (Table 2). Extrapolated values from the standard curves indicate percentages ranging from 8.314 × 10^−3^–3.6 × 10^−5^%, although these values are unreliable, given that they fall outside the standard curve.

**Table 2:** Details quantitative PCR for each CB that came up positive in the screening.

Case No.	No. systems tested	No. wells that came up positive	Maternal chimerism confirmed?	% maternal DNA
3	1	0 of 5	No	
5	1	3 of 5	Yes	<0.1
	2	5 of 5		<0.01
6	1	1 of 5	No	
7	1	0 of 5	No	
13	1	4 of 5	Yes	<0.1
16	1	5 of 5	Yes	<0.1
17	1	1 of 5	No	
19	1	1 of 5	No	
21	1	4 of 5	Yes	<0.01
22	1	0 of 5	No	
	2	1 of 5	No	
25	1	1 of 5	No	
26	1	1 of 5	No	
	2	5 of 5	Yes	<0.01
28	1	1 of 5	No	
30	1	1 of 5	No	
31	1	5 of 5	Yes	<0.01
32	1	2 of 5	No	
34	1	0 of 5	No	
35	1	0 of 5	No	
36	1	0 of 5	No	
37	1	3 of 5	Yes	<0.01
39	1	1 of 5	No	

Using these non-stringent criteria, we found that only 17.9% of the CBs that we tested showed any sign of containing maternal DNA, and when it was detected, its abundance was too low for reliable quantification.

Given the potential importance of maternal T cells in particular in CB transplantation (as opposed to other leukocytes), we considered the possibility that the frequency of maternal cells within the T cell population in CB might be higher than that in the total PBMC fraction. For that reason, we performed separate qPCRs on purified CD4^+^ and CD8^+^ T cells in two samples previously found to contain detectable maternal DNA. Even after this preselection and with a high input of DNA (300–500 ng per sample), the abundance of maternal DNA remained undetectable in the CD4^+^ fraction and below 0.001% in the CD8^+^ fraction in one of the samples. Based on both cell-based and DNA-based methods, we therefore conclude that the prevalence of maternal T cells in CBs is very low in our cohort of CBs.

## Discussion

Correlative evidence based on HLA-matching has indicated that maternal T cells in CB may positively affect the outcome of CB transplantation in leukaemia patients^6^. There has, however, been no conclusive evidence that maternal IPA-reactive T cells are indeed present in CB and able to exert bona fide anti-leukaemic effector functions. Some studies did report relatively high numbers of maternal cells in CB and CB-origin MMc could still be detected in a patient up to 6 months post-transplantion^18^, lending credence to the idea that such cells could impact the outcome of transplantation therapy^7–9^. Quantification of maternal T cells in CB might thus be a useful criterion (in addition to HLA-matching) to select CBs for transplantation. We therefore compared four different approaches to detect maternal T cells in CB samples. Three of these relied on FACS-based detection of intact cells, as was done in the landmark study by Mold, *et al*.^7^. The frequencies of maternal T cells that we managed to detect through FACS-based methods were much lower than those reported in that study. In fact, we could not corroborate that the few cells detected were truly of maternal origin, as highly efficient enrichment methods (positive selection via MACS or FACS with subsequent culture of the sorted cells) failed to increase their frequency. Reasons for false assignment of maternal identity to cells in CB may relate to signal noise and, as we have found, asymmetric loss of HLA expression after storage of CB. Especially the differences between the stability of different HLA molecules poses a challenge for reliable detection of MMc. Downregulation of HLA molecules is exacerbated by delays in analysis, but may be a problem even when analysing samples early on: the low frequencies of cells that must be detected make even limited loss of HLA expression a significant factor. For these reasons, we also used a fundamentally different detection method based on measurement of maternal DNA. Through qPCR using In/Del polymorphisms, we found evidence for MMc in fewer than 20% of CBs, and in those samples, the abundance of maternal DNA remained well below the quantifiable limit.

There is convincing evidence from mice and human abortion material that maternal cells can transfer to the foetus during the first and second trimester of pregnancy^19–21^. Several studies have reported the presence of nuclei^8,9^ or DNA of maternal origin in CB^11–15^. However, only one study has shown the presence of intact maternal cells in full-term CB^7^, a finding that we could not replicate. Our results are consistent with a study, in which a very sensitive PCR-based method (0.01-0.001%) only detected maternal DNA in less than a quarter of full-term CBs^10^. The lower detection threshold of our FACS-based detection method was around 0.001%. Our inability to unequivocally detect viable maternal T cells in CB by this method would therefore suggest that such a CB graft, when administered at 2,5 * 10^7^ nucleated cells/kg body weight of the recipient (official FDA guidelines, section 2.1)^22^, contains fewer than 250 maternal nucleated cells/kg recipient body weight. Taking into consideration the efficacy of our MACS enrichment (two orders of magnitude), the true frequency of such cells in the samples analysed was probably much lower still. Even if all maternal cells in the CB would be T cells, the total number of such cells would presumably amount to no more than a few hundred cells per transplantation at most. To influence the outcome of stem cell transplantation, such cells would have to be remarkably potent. Although this is not impossible, it is important to take into consideration that there is great variation in the percentages of maternal cells in CB (ranging from 0.004–1%) that have been reported^11–15^. Perhaps therefore, the effect found from IPA-matching between CB and transplant recipient^6^ stems from those CBs with relatively high frequencies. One explanation for the large differences in frequencies of maternal T cells in CB reported by different groups, could be that these cells are introduced into the graft during the specific harvesting procedure used to obtain the CB graft. It has been suggested that labour itself may contribute to materno-foetal exchange of cells^23,24^, which could lead to differences in MMc between CB harvested after vaginal delivery or after caesarean section. Another possible factor that could influence maternal cell numbers in CB grafts is the time of harvesting. Some CB banks (including the one from which the samples in the present study were obtained) collect their samples when the placenta is still *in utero*^25^ and others harvest their CB after placental delivery^26,27^. In the latter case, it is conceivable that contractile expulsion of the placenta could force maternal cells, present in the blood vessels and sinusoids, into the foetal compartment of the CB.

In conclusion, our study shows that maternal T cells are present at very low frequencies if at all, at least in the more than 50 CBs tested in our cohort. As the presence of maternal IPA-specific T cells may have very important therapeutic benefits, it might be worthwhile to select CBs with a high frequency of maternal T cells for transplantation. To facilitate this screening, it is important to determine the reason why some CBs have higher frequencies of maternal T cells than others. Furthermore, given the caveats from FACS-based detection methods, such analysis would preferentially include a qPCR-based detection method.

## Methods

### Blood samples

Umbilical cord blood was obtained from full-term infants of healthy mothers who went through pregnancy without complications. After informed consent from the mother, 40–80 ml of CB was collected into a precitrated collection bag with the placenta still *in utero*. Blood from the mother was also collected in EDTA-coated tubes. CB was obtained from the Sanquin cord blood bank, according to the Eurocord guidelines. Whole blood samples or buffy coats from healthy anonymised donors were obtained after their written informed consent. All human materials were obtained in accordance with the Declaration of Helsinki and the Dutch rules and regulations with respect to the use of human materials from volunteer donors, as approved by Sanquin’s internal ethical board. Peripheral and cord blood mononuclear cells (PBMCs/CBMCs) were isolated up to 72 h after sample harvesting, using a Ficoll-Paque Plus (GE Healthcare) gradient. For long-term storage in liquid nitrogen, samples were frozen in 10% DMSO in foetal calf serum (FCS).

### Antibody staining and FACS analysis

Cells were incubated with FcR Blocking Reagent (Miltenyi) according to the manufacturer’s instructions prior to staining with anti-HLA antibodies. Surface staining of cells was done in PBS containing 0.5% FCS for 15 min. at room temperature. To allow for the exclusion of dead cells from the analysis, a Live/Dead marker (DAPI (Sigma, D9564), 7-AAD (Beckman Coulter, A07704), To-Pro3 (Invitrogen, T3605) or NearIR (ThermoFisher, L10119)) was always included in the staining. Expression levels of all markers were measured using an LSR II cytometer (BD Biosciences), and data were analysed using the FlowJo software (version 10; Tree Star). The following monoclonal antibodies (mAbs) against human HLAs were used: GV2D5 (anti-HLA-A*01), SN607D8 (anti-HLA-A*02/28), OK2F3 (anti-HLA-A*03), VTM1F11 (anti-HLA-B*07/27/60), BVK1F9 (anti-HLA-B*08), DK7C11 (anti-HLA-B*12/44/45), HDG8D9 (anti-HLA B*35/51). All of these were developed in our laboratory^28^. Antibodies were produced in stirred-tank format, purified and labelled with fluorochromes as previously described^29^. For determining the maternal cell gate, PBMCs from the mother were stained with CellTrace™ Violet (Thermo Fischer Scientific, C34557) according to the manufacturer’s instructions and mixed into CB aliquots before anti-HLA antibody staining.

### MACS pre-enrichment

Surface staining for maternal HLA was performed as described above with PE-labelled anti-HLA antibodies. Cells were then washed with MACS buffer (0.5% FCS in PBS) and incubated with anti-PE MicroBeads (Miltenyi, 130-048-801) in a 1:5 dilution in MACS buffer and incubated for 20 min. at 4 °C. After incubation, the cells were again washed and passed over an LS column (Miltenyi) according to the manufacturer’s instructions. The enriched fraction of the column was then stained for other surface markers as described above.

### Cell sorting and culture

Prior to culture, cells were sorted on a BD FACSAria III (BD Biosciences), using the same method for determining the maternal cell gate as described for FACS analysis. Cells were then plated at +/−20.000 cells/well in a 96-wells round bottom plate (Greiner Bio, 650161) and cultured in IMDM +10% FCS +1% L-Glut +1% P/S for 24 days in the presence of 300 IU/ml IL-2 (Proleukin; Novartis, 730525). On day 0, cells were stimulated with soluble anti-CD3 (0,1 µg/ml; M1654, clone 1XE, PeliCluster) and anti-CD28 (0,1 µg/ml; 16-0289-85, clone CD28.2, eBioscience). Fresh medium with 300 IU/ml IL-2 was added on day 4, 11 and 14 of culture. On day 7 and 21 cells were counted where possible and split to the original starting concentration of 20.000 cells/well (96-wells roundbottom plate) or 500.000 cells/well (24-wells plate) before restimulation with anti-CD3/anti-CD28 and IL-2 at the same concentrations as listed above.

### DNA isolation, In/Del screening and qPCR

Genomic DNA was isolated from 50 CB and paired MB mononuclear cell samples using the QIAamp DNA Blood Mini kit (Qiagen) according to the manufacturer’s instructions. MMc was analysed through 13 specific biallelic short insertion/deletion (In/Del) polymorphisms (S03, S04a, S04b, S05b, S06, S07a, S07b, S08b, S09b, S10b, S11b, MID836b, MID847b)^17,30^. These biallelic systems allow discrimination between samples from two different subjects in 90% of pairs and have a reproducible sensitivity of detecting at least 0.1% chimerism at an input of 500 ng DNA. We pre-screened to identify In/Del systems for which the mother of the CB/MB pair was positive, but the child was negative. For this purpose, we ran all systems in duplicate on all 100 samples with an input of 2,25 ng DNA. Next, we ran a qPCR with the max input of CB (~500 ng) DNA in triplicate for up to 3 systems for which only the mother was positive in order to see if any maternal DNA could be detected in that sample. For all the samples where at least one well came up positive for a system, that system was then quantitatively analysed with a standard curve for 5 replicates of the CB sample (~500 ng DNA input) in order to define the magnitude of the MMc. MMc was confirmed when at least 3 of the 5 wells came up positive and the percentage of MMc was then deduced from the standard curve and based on the sensitivity of that system (lowest concentration in standard curve where both wells still had a CT-value).

## Supplementary information

Supporting Online Material

## Data Availability

The data generated during and/or analysed during the current study are available from the corresponding author on reasonable request.

## References

[1] Munoz-Suano A, Hamilton AB, Betz AG (2011). Gimme shelter: The immune system during pregnancy. Immunol. Rev..

[2] Van Kampen, C. A. *et al*. Pregnancy can induce long-persisting primed CTLs specific for inherited paternal HLA antigens. *Hum*. *Immunol*., 10.1016/S0198-8859(01)00209-9 (2001). 10.1016/s0198-8859(01)00209-9 11250037

[3] Verdijk, R. M. *et al*. Pregnancy induces minor histocompatibility antigen-specific cytotoxic T cells: Implications for stem cell transplantation and immunotherapy. *Blood*, 10.1182/blood-2003-05-1625 (2004). 10.1182/blood-2003-05-1625 14592836

[4] Mommaas B (2005). Cord blood comprises antigen-experienced T cells specific for maternal minor histocompatibility antigen HA-1. Blood.

[5] Dalle JH (2004). Results of an unrelated transplant search strategy using partially HLA-mismatched cord blood as an immediate alternative to HLA-matched bone marrow. Bone Marrow Transplant..

[6] van Rood JJ, Scaradavou A, Stevens CE (2012). From the Cover: Indirect evidence that maternal microchimerism in cord blood mediates a graft-versus-leukemia effect in cord blood transplantation. Proc. Natl. Acad. Sci..

[7] Mold JE (2008). Maternal alloantigens promote the development of tolerogenic fetal regulatory T cells in utero. Science.

[8] Hall JM (1995). Detection of maternal cells in human umbilical cord blood using fluorescence *in situ* hybridization. Blood.

[9] Wernet P, Kögler GST (1994). The rapid detection of the quantity (genotype) and cell lineage (immunophenotype) of contaminating maternal white cells in cord blood samples by fluorescence *in situ* hybridization combined with confocal laser scanning microscopy. Blood Cells.

[10] Roh EY (2017). Frequency of fetal-maternal microchimerism: an analysis of the *HLA-DRB1* gene in cord blood and maternal sample pairs. J. Matern. Neonatal Med..

[11] Lo YMD, Lau TK, Chan LYS, Leung TN, Chang AMZ (2000). Quantitative analysis of the bidirectional fetomaternal transfer of nucleated cells and plasma DNA. Clin. Chem..

[12] Morin AM (2017). Maternal blood contamination of collected cord blood can be identified using DNA methylation at three CpGs. Clin. Epigenetics.

[13] Scaradavou A, Carrier C, Mollen N, Stevens C, Rubinstein P (1996). Detection of maternal DNA in placental/umbilical cord blood by locus-specific amplification of the noninherited maternal HLA gene. Blood.

[14] Poli F (1997). Detection of maternal DNA in human cord blood stored for allotransplantation by a highly sensitive chemiluminescent method. J Hematother.

[15] Briz M (1998). Detection of maternal DNA in umbilical cord blood by polymerase chain reaction amplification of minisatellite sequences. Bone Marrow Transplant..

[16] Moon JJ (2007). Naive CD4+ T Cell Frequency Varies for Different Epitopes and Predicts Repertoire Diversity and Response Magnitude. Immunity.

[17] Alizadeh M (2002). Quantitative assessment of hematopoietic chimerism after bone marrow transplantation by real-time quantitative polymerase chain reaction. Blood.

[18] Kanaan SB (2017). Maternal microchimerism is prevalent in cord blood in memory T cells and other cell subsets, and persists post-transplant. Oncoimmunology.

[19] Jonsson, A. M., Uzunel, M., Götherström, C., Papadogiannakis, N. & Westgren, M. Maternal microchimerism in human fetal tissues. *Am*. *J*. *Obstet*. *Gynecol*. **198** (2008). 10.1016/j.ajog.2007.09.047 18191801

[20] Götherström C, Johnsson AM, Mattsson J, Papadogiannakis N, Westgren M (2005). Identification of maternal hematopoietic cells in a 2nd-trimester fetus. Fetal Diagn. Ther..

[21] Kinder JM (2015). Cross-Generational Reproductive Fitness Enforced by Microchimeric Maternal Cells. Cell.

[22] HPC, Cord Blood Injectable Suspension for Intravenous Use. Available at, https://www.fda.gov/downloads/BiologicsBloodVaccines/CellularGeneTherapyProducts/ApprovedProducts/UCM305761.pdf.

[23] Buxmann H (2016). Maternal CD4+ microchimerism in HIV-exposed newborns after spontaneous vaginal delivery or caesarean section. Early Hum. Dev..

[24] Masuzaki H (2004). Labor increases maternal DNA contamination in cord blood [6]. Clinical Chemistry.

[25] Lauber S, Latta M, Klüter H, Müller-Steinhardt M (2010). The Mannheim Cord Blood Bank: Experiences and Perspectives for the Future. Transfus. Med. Hemother..

[26] Davey S (2004). The London Cord Blood Bank: Analysis of banking and transplantation outcome. Br. J. Haematol..

[27] Querol S (1998). The placental blood program of the Barcelona Cord Blood Bank. Bone Marrow Transplant..

[28] Mulder A (2010). Human monoclonal HLA antibodies reveal interspecies crossreactive swine MHC class I epitopes relevant for xenotransplantation. Mol. Immunol..

[29] Van Hensbergen Y, Mulder A, Cornelissen JJ, Brand A (2013). Validation of human monoclonal HLA Class i antibodies to evaluate the kinetics of donor chimerism in different cell subsets after double-cord-blood transplantation in the NOD/SCID model. Transfusion.

[30] Scheffer PG (2010). Reliability of fetal sex determination using maternal plasma. Obs. Gynecol.

