# Supplementary material for: Determining the extent of maternal-foetal chimerism in cord blood
Source: Sci Rep. 2019 Mar 27;9:5247. doi: 10.1038/s41598-019-41733-w (PMC6437214; doi:10.1038/s41598-019-41733-w)
Supplement: Supplementary file 1 — Supporting Online Material [file 41598_2019_41733_MOESM1_ESM.pdf]

Supporting Online Material for

Determining the extent of maternal-foetal chimerism in cord blood

Rianne Opstelten, Manon C Slot, Neubury M Lardy, Arjan C Lankester, Arend Mulder, Frans HJ Claas, Jon J van Rood, Derk Amsen*

*To whom correspondence should be addressed. E-mail: d.amsen@sanquin.nl

This PDF file includes:

- Methods
- Supplementary Figure S1
- References

SUPPLEMENTARY METHODS

Blood samples

Blood from healthy anonymised donors was obtained after their written informed consent. All human materials were obtained in accordance with the Declaration of Helsinki and the Dutch rules and regulations with respect to the use of human materials from volunteer donors, as approved by Sanquin's internal ethical board. Peripheral mononuclear cells were isolated up to 72h after sample harvesting, using a Ficoll-Paque Plus (GE Healthcare) gradient.

Antibody staining and FACS analysis

The following monoclonal antibodies (mAbs) against human HLAs were used: VTM1F11 (anti-HLA-B7/27/60, PE labelled), BVK1F9 (anti-HLA-B8, AF488 labelled), Sanbio BIH1453 (anti-HLA-B7/27, biotin labelled) and Sanbio BIH0536A (anti-HLA-B8, biotin labelled). VTM1F11 and BVK1F9 were developed in our laboratory[1,2]. Cells were stained with anti-HLA antibodies in combination with FcR Blocking Reagent (Miltenyi). Surface staining of cells was done in PBS containing 0.5% FCS for 15 min. at room temperature. After staining with biotin-labelled HLA-antibodies, a subsequent staining with streptavidin (PerCp-Cy5.5 labelled (BD Pharmingen, 551419)) was done in PBS containing 0.5% FCS for 15 min. at room temperature. To allow for the exclusion of dead cells from the analysis, a Live/Dead marker (NearIR (ThermoFisher, L10119)) was included in the staining. Expression levels of all markers were measured using an LSR II cytometer (BD Biosciences), and data were analysed using the FlowJo software (version 10; Tree Star).

SUPPLEMENTARY FIGURE S1

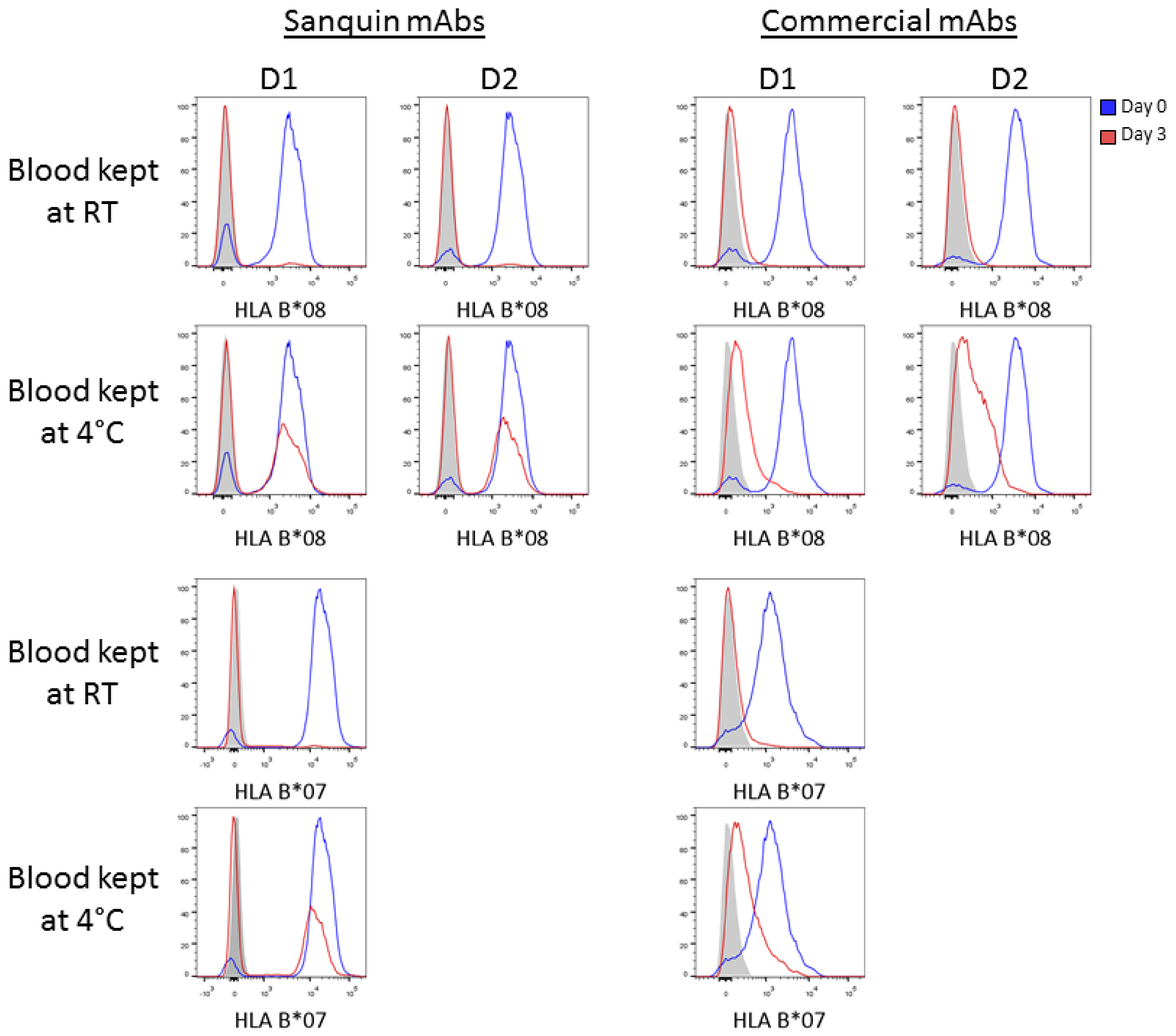

Supplementary Figure S1. Storage-related downregulation of HLA expression is not due to changes in specific HLA-epitopes. Whole blood in EDTA from two donors (D1 HLA B*07/08 & D2 HLA B*08/08) was either kept at room temperature (RT) or at 4°C for 3 days. Blood was Ficolled on the day of the HLA-staining and FACS analysis. Modal histograms per donor (column) and per HLA-antibody (rows) are shown comparing the staining on Day 0 (blue) to that on Day 3 (red) against the background (grey) for antibodies generated in our laboratory (left) and commercially available antibodies (right).

SUPPLEMENTARY REFERENCES

1. Mulder, A. *et al.* Human monoclonal HLA antibodies reveal interspecies crossreactive swine MHC class I epitopes relevant for xenotransplantation. *Mol. Immunol.* **47,** 809–815 (2010).

2. Van Hensbergen, Y., Mulder, A., Cornelissen, J. J. & Brand, A. Validation of human monoclonal HLA Class i antibodies to evaluate the kinetics of donor chimerism in different cell subsets after double-cord-blood transplantation in the NOD/SCID model. *Transfusion* **53,** 104–114 (2013).
